# Differences in Biometric Fetal Weight Estimation Accuracy and Doppler Examination Results in Uncomplicated Term Singleton Pregnancies between Vertex and Breech Presentation

**DOI:** 10.3390/jcm10153252

**Published:** 2021-07-23

**Authors:** Lukas Jennewein, Simon Theissen, Hemma Roswitha Pfeifenberger, Nadja Zander, Kyra Fischer, Christine Eichbaum, Frank Louwen

**Affiliations:** 1 Department of Gynecology and Obstetrics, School of Medicine, Goethe-University, 60590 Frankfurt, Germany; simon_theissen@web.de (S.T.); HemmaRoswitha.Pfeifenberger@kgu.de (H.R.P.); Kyra.Fischer@kgu.de (K.F.); Christine.Eichbaum@kgu.de (C.E.); louwen@em.uni-frankfurt.de (F.L.); 2 Carl Remigus Medical School, 65510 Idstein, Germany; Nadja.Zander@web.de

**Keywords:** doppler, breech, fetal weight estimation

## Abstract

Doppler examination of the umbilical artery and the fetal middle cerebral artery is evaluated predominantly in pregnancies with fetuses in cephalic presentation and never has been elucidated in breech presentation. Evidence on the accuracy of fetal weight estimation in dependence of the fetal presentation is controversial. Nevertheless, clinical decisions including recommendations for a cesarean section or labor induction based on these examinations are applied to pregnancies with fetuses in breech presentation. The objective of this study was to investigate the influence of the fetal presentation on fetal weight estimation accuracy, umbilical artery and middle cerebral artery resistance indices (RI) in a prospective case control study. Ultrasound examinations in 305 uncomplicated term pregnancies (153 vertex presentations, 152 breech) were investigated. Non-parametric variables were compared using Pearson’s chi^2^ test and Wilcoxon chi^2^ test, depending on variable scaling. Fetal weight estimation accuracy was not significantly different between vertex presentation group (VP) (6.97%) and breech presentation group (BP) (7.96%, *p* = 0.099). Fetal head circumference measurements were significantly larger in BP (350 mm vs. 341 mm in VB, *p* > 0.0001) while abdominal circumferences were significantly smaller (VP: 338 mm, BP: 331 mm, *p* = 0.0039) and weight estimation was not significantly different. Umbilical artery RIs were not significantly different between VP (54.5) and BP (55.3, *p* = 0.354). Fetal middle cerebral artery RIs also showed no significant differences (VP: 71.2, BP: 70.7, *p* = 0.335). Our study shows that fetal Doppler (RI) and weight estimation ultrasound originally calibrated in cephalic pregnancies are applicable to pregnancies with fetuses in breech presentation.

## 1. Introduction

Doppler and biometric ultrasound is a helpful tool during and at the end of each pregnancy. Fetal growth is observed throughout the pregnancy in order to detect growth restriction or macrosomia. Doppler ultrasound of the fetal umbilical cord and the fetal middle cerebral artery are important parameters for fetal observation, especially in cases with fetal growth restriction [1,2,3]. Fetal examination with weight estimation and Doppler ultrasound generates obstetric clinical measures, as stated in the national guidelines [4,5,6]. In the national guidelines on breech presentation management, fetal weight estimation is an important issue as well because, e.g., the British guideline requires a fetal weight of 2.5 kg–3.5 kg to recommend a vaginal birth approach [7]. Another requirement is to ensure that no fetal compromise is apparent prior to birth [7]. Enhanced surveillance, labor induction and even immediate cesarean section are direct consequences resulting from suspect or pathological Doppler examinations [8]. Resistance index (RI) reference ranges were established mostly in vertex pregnancies and/or there was no comment on the fetal presentation [9,10]. Studies analyzing Doppler indices use cohorts with both cephalic and breech presenting fetuses. Until today these RI and biometric weight estimation cut offs are used also in cases with fetuses in breech presentation. Here, pressure conditions, e.g., on the fetal head, might be different from vertex presentation because the pressure of the pelvis, forming the fetal head before birth does not apply. Head circumference measurements during biometric weight estimation thus might be altered by fetal positioning [11,12]. Melamed et al., showed that in vertex presentation fetal head circumference estimation is quite inaccurate [13]. There is only little known about differences in Doppler and biometric examination results depending on the fetal presentation. Only one study examined fetal circulation taking the fetal presentation into account, namely before and after external cephalic version. There were no significant differences [14]. Fetal weight estimation accuracy in relation to the fetal presentation has been investigated—with contradicting results. In a study by McNamara et al. [15] and another by Dammer et al. [16] no differences in biometric accuracy was found, while Melamed et al. [17] and Shmueli et al. [18] detected a lower accuracy in fetal weight estimation in breech presentation compared to vertex presentation. Knowledge of significant discrepancies and differences in estimation failures in uncomplicated pregnancies might lead to an adaption of known reference ranges of biometric parameters (head circumference, abdominal circumference, and femur length) and resistance indices of the umbilical and middle cerebral artery in pregnancies with breech presentation. This would have direct clinical impact since clinical management recommendations are based on these examinations. We hypothesize that fetal biometric weight measurement might be less accurate but Doppler estimations should show similar results in breech presented fetuses compared to fetuses in vertex presentation. In order to detect differences in uncomplicated pregnancies regarding sonographic biometrics and Doppler examination at the end of pregnancy, we conducted a prospective case-control study on 330 patients.

## 2. Materials and Methods

### 2.1. Patient Cohort and Ethics Approval

This is a prospective case control study. From January 2016 until February 2019, 165 uncomplicated singleton primipara pregnancies with breech presentation and 165 pregnancies with cephalic fetal presentation were included. Matching features were mothers age and BMI. Included were only pregnancies with delivery after 36 + 6 weeks of pregnancy. Excluded were complicated pregnancies, for example preeclampsia, IUGR and fetal malformations. The standard hospital care provides Doppler and weight estimation examination to each patient who is admitted to birth since the majority of patients have complicated pregnancies. Therefore, also women with uncomplicated pregnancies are offered the same treatment. Ethics approval was received from the ethics committee of the Goethe University Hospital in Frankfurt, Germany with the reference number 19–332. Informed patient’s consent was not required because data was obtained after patients discharge and only standard clinical procedure was observed. Data was collected anonymized from the hospital’s patient documentation system.

### 2.2. Ultrasound Examination and Clinical Protocol

In order to detect differences in biometrical accuracy and Doppler-sonografically obtained resistance indices (RI) of the fetal umbilical artery (ateria umbilicalis, AU) and the fetal middle cerebral artery (arteria cerebri media, MCA), 330 pregnant women at term (165 breech, 165 vertex) were included between January 2016 until February 2019. Ultrasound was performed in accordance to up to date recommendations by qualified obstetricians (in total 36 clinicians) at admission to the hospital with identical methodology (free floating piece of the umbilical cord, angle correction of the middle cerebral artery, and correct anatomic section on pictures of fetal weight estimation). If ultrasound examinations were performed by an intern, the examination was supervised and/or repeated by a specialist (supervising physician). Pictures from these examinations were checked for quality control (measurements, appropriate depiction) from the authors of this study. Measurements were not blinded. Ultrasound examinations and following birth-related variables were documented. Because the cohort of cephalic singleton pregnancies was immensely larger, we needed to select a representative sub-cohort. Out of 2333 patients meeting the inclusion criteria (primipara, vertex presentation, no fetal growth restriction, no preeclampsia, no prematurity), 165 cases were automatically and randomly picked using JMP 14.0 software (SAS, Heidelberg, Germany). There were no significant differences in the two resulting groups regarding parity, BMI, age, and duration of pregnancy (see Table 1). Due to incomplete data, 25 patients were excluded. Ultrasound examinations were performed by obstetricians before labor using a GE Voluson P8 50/60 Hz ultrasound (GE Healthcare, Solingen, Germany) with pulsed-wave color-coded Doppler function. RI values were obtained automatically. Biometric fetal weight estimation was performed using Hadlock formula after measuring fetal head circumference and biparietal diameter, fetal abdominal circumference, and fetal femur length. To calculate estimation inaccuracy, we subtracted actual birth weight and estimated birth weight for each measurement. Δbirth weight was divided by birth weight to gain relative birth weight measurement discrepancy.

### 2.3. Statistical Analysis

Statistical analysis was performed using JMP 14 Software from SAS. Normal distribution was tested using Kolmogorov–Smirnov test and did not apply for all variables. There were no outliers. For continuous variables, *t*-test was performed to detect differences. For non-parametrical analysis of differences, Pearson’s Chi^2^ testing was performed on nominal scales variables. Differences of continuous variables (interval scaled, e.g., RI values) were tested with Wilcoxon chi^2^ test. A *p*-value of below 0.05 (alpha) was regarded as significant.

## 3. Results

Vertex presentation group and breech presentation group did not differ in mothers age, BMI, fetal sex or fetal birth weight as shown in Table 1. Cesarean section rate was significantly higher in the breech presentation group (54.6%) in comparison to cephalic deliveries (24.8%) (Table 1).

When the sonographic findings were analyzed, it should be noted that the time point of examination was at 40th week of pregnancy and the timepoint did not differ between groups significantly (Table 2). There were 25 cases with insufficient documentation which lead to exclusion. The fetal back orientation as well as the placenta location was not significantly different between groups. Biparietal diameter was significantly larger in breech presentations (BP) as compared to vertex presentations (VP) (BP: 97.7 vs. VP: 96.4 mm, *p* = 0.0438) (Table 2). Head circumference also was significantly larger in breech presentations (BP: 349.9 vs. VP: 341.1 mm, *p* > 0.0001). Abdominal circumference (VP: 337.9 vs. BP: 331.3 mm, *p* = 0.0049) and femur length (VP: 75.9 vs. BP: 75.2 mm, *p* = 0.0342) measurements were significantly larger in VB (Table 2). Calculated fetal weight estimation did not differ significantly between groups (VP: 3486 g vs. BP: 3443 g, *p* = 0.182) (Table 2).

Resistance indices of the umbilical artery were not significantly different with a value of 54.5 in VP and 55.3 in BP group, *p* = 0.354. RI of the middle cerebral artery also was not significantly different in VP (71.2) as compared to BP group (70.7, *p* = 0.335) (Table 2).

To examine the accuracy of estimated fetal weight, differences in estimation and fetal birth weight were calculated. Mean difference in grams was 238 g in VP group and 269 g in BP group. The difference was not significant. (*p* = 0.135, Table 3). Percentage of estimation failures were 6.97% in VP group and 7.96% in BP group, showing a not significant tendency (*p* = 0.099) to a greater inaccuracy in birth weight estimation in the breech presentation group (Table 3).

## 4. Discussion

Until today there was almost no data comparing Doppler examination values and only controversial evidence comparing biometric fetal weight estimation in breech and cephalic presentation. In this study of 305 uncomplicated pregnancies, we examined fetal weight estimation and resistance indices of the umbilical artery and the fetal middle cerebral artery in dependence of fetal presentation.

Resistance indices of the umbilical artery were not significantly different between vertex and breech presentation in uncomplicated pregnancies (Table 2). This is in line with the results of Lau et al. [14]. They compared fetal circulation before and after external cephalic version. The resistance indices of the fetal middle cerebral artery were not different in respect to the fetal presentation (Table 2). Leung et al. [19] report differential blood flow after external cephalic version but refer this effect to force executed during the procedure and not the fetal presentation. Blood circulation is protected by vasoactive hormones and muscular vessels. A significant alteration in fetal blood flow through positioning within the uterus is unlikely.

With our data we cannot recommend any alterations of reference ranges of resistance indices in breech pregnancies. National guidelines requiring signs of fetal wellbeing [7,20] can be enabled. Doppler examination is established for fetal surveillance in growth restriction. This study was performed on uncomplicated pregnancies. When placenta insufficiency is detectable through Doppler examination, fetal presentation should not have an impact on those values. Presumably, measurements in earlier weeks of gestation are not likely to be affected by fetal presentation either.

Fetal weight estimation with a mean inaccuracy of 6.97% (VP) and 7.96% (BP) overall was quite low (Table 3). A systematic review from Milner et al. [21] reports improving estimation accuracy over time (below 10%). Our results are in line with that. Difference of estimation accuracy in our analysis was not significant. Estimated mean weight between groups was not different. Abdominal circumference and femur length mean estimations were significantly larger in VP than in BP group (Table 2). These differences might be explained by fetal posture. In the majority of cases, fetuses in breech presentation are in frank breech presentation, with their legs stretched next to their abdomen. Femur length is more difficult to display. Resulting compression of the fetal abdomen might falsely lead to lower abdominal circumference measurements. In our study, newborns out of breech presentation have a greater head circumference compared to fetuses born out of vertex position (Table 3). It is known that fetuses in breech presentation have a more dolichocephalic head shape [12], leading to larger head measurements and explaining our data. This could be explained by pressure mediated overlapping skull bones in vertex positioned infants. Of note, even though statistical significance applies for some parameters, the clinical impact is limited with only few millimeters of difference between groups. Furthermore, our study does not show a significant difference in fetal weight estimation accuracy depending on the fetal presentation, even though there is a non-significant tendency towards a larger inaccuracy in breech presentations which might be overcome through enlarging the cohort. Thus, our results are in line with McNamara et al. [15] and Dammer et al. [16] who found no differences in weight estimation accuracy. Fetal weight estimation is an important examination in order to determine a recommendation for a vaginal birth approach. It is required in national breech guidelines: The British green top guideline states that vaginal birth approach is feasible when weight estimation is not above 3500 g [7]. In the American guideline, the required birth weight estimation is 2500–4000 g [22]. Louwen et al., showed an over-all good fetal outcome in vaginal breech deliveries comparable with cephalic deliveries in an upright birth position independent from birth weight [23]. In another study comparing mortality rates in vaginally attempted breech deliveries between fetuses weighing more or less than 3800 g no difference in fetal outcome was shown [24]. Of note, cesarean section rates increase with growing birth weight—which is an important issue to discuss within patients counselling. Birth weight estimation in breech presentation consequently has similar importance and purpose as in cephalic pregnancies. It is crucial to use applicable thresholds, formulas, and reference margins. Otherwise, cesarean section is recommended to patients who might have had a chance to deliver vaginally which carries severe perioperative risks. Our provided data does not suggest the need of reference range adjustment or biometric formula revision because mean estimation inaccuracy in breech presentations was below 10%.

A strength of our study is the quite large number of cases with breech presentation and an equal sample size with a matched control group. In this study only the resistance index is analyzed. S/D ratio, which is important for clinical implications can be calculated with either resistance index or pulsatility index (PI). The PI is the most common used index to evaluate fetal blood flow and was not evaluated in this study. Thus, reproducibility might be limited for some obstetrical centers.

## 5. Conclusions

Our study provides evidence that examinations including fetal Doppler (RI) and weight estimation ultrasound originally established in pregnancies with fetuses in vertex presentation are applicable to pregnancies with fetuses in breech presentation. Chances in reference ranges of umbilical artery RI, fetal middle cerebral artery RI, or Hadlock biometrical fetal weight estimation are not necessary in order to maintain high quality of clinical practice in uncomplicated term pregnancies. Obstetricians should be aware of the limitations of their examinations and especially of the inaccuracy of fetal weight estimation in both breech and vertex presentation.

## Figures and Tables

**Table 1:** General variables of pregnancies in vertex position group and breech presentation group.

Variables	Vertex Presentation *n* = 153	Breech Presentation *n* = 152	*p* Value
Mothers Age [mean ± std. dev., years]	31.4 (± 5.5)	31.7 (± 3.7)	0.548
Mother height [mean ± std. dev., cm]	168 (± 5.81)	168 (± 6.43)	0.830
Mother weight [mean ± std. dev. kg]	63 (± 8.33)	64 (± 8.54)	0.689
Mother BMI Mutter [mean ± std. dev. kg/m^2^]	22.5 (± 2.8)	22.6 (± 2.6)	0.690
Pregnancy duration [mean ± std. dev. d]	40.4 (± 0.6)	40.5 (± 0.6)	0.054
Vaginal birth	115 (75.2%)	69 (45.4%)	<0.0001
Cesarean section	38 (24.8%)	83 (54.6%)	<0.0001
Fetal sex: male	70 (45.8%)	71 (46.7%)	0.867
Fetal sex: female	83 (54.3%)	81 (53.3%)	0.867
Birth weight [mean ± std. dev., g]	3439 (± 367)	3378 (± 378)	0.185

**Table 2:** Ultrasound findings and measurements of Doppler sonography and biometric fetal weight estimation in vertex presentation and breech presentation group.

Variables	*n* (Σ)	Vertex Presentation	Breech Presentation	*p* Value
Timepoint of examination [mean, week of pregnancy]	305	40.0 (±0.58)	40.1 (±0.53)	0.075
Fetal orientation				0.372
Fetal back on the left side	121	54 (68.4%)	67 (62.0%)	
Fetal back on the right side	66	25 (31.7%)	41 (38.0%)	
Placenta location				0.902
Dorsal placenta	99	48 (35.8%)	51 (38.6%)	
Ventral placenta	135	71 (53.0%)	64 (48.5%)	
Cranial placenta	11	5 (3.7%)	6 (4.6%)	
Lateral placenta	21	10 (7.5%)	11 (8.3%)	
Biparietal diameter [mean, mm ± std. dev.]	264	96.4 (±3.97)	97.7 (±4.33)	0.0438
Head circumference [mean, mm ± std. dev.]	264	341.1 (±11.73)	349.9 (±11.57)	<0.0001
Abdominal circumference [mean, mm ± std. dev.]	263	337.9 (±18.58)	331.3 (±18.52)	0.0039
Femur length [mean, mm ± std. dev.]	263	75.9 (±2.97)	75.2 (±3.14)	0.0342
Estimated fetal weight [mean, g ± std. dev.]	264	3486 (±365)	3443 (±381)	0.182
RI fetal A. umbilicalis [*%*, mean ± std. dev.]	288	54.5 (±6.1)	55.3 (±6.1)	0.354
RI fetal A. cerebri media [*%*, mean ± std. dev.]	239	71.2 (±6.5)	70.7 (±7.1)	0.335

Loss of data was due to incomplete documentation or biometric fetal weight estimation in a prior examination more than 1 week before delivery.

**Table 3:** Estimated and actual birth weights and estimation failures/differences (calculated with values).

Variables	Vertex Presentation	Breech Presentation	*p* Value
Birth weight [mean ± std. dev., g]	3439 (±366)	3378 (±378)	0.185
Estimated fetal weight [mean ± std. dev., g]	3486 (±365)	3443 (±381)	0.182
Total estimation failure [mean ± std. dev., g]	238.2 (±183)	269.3 (±188)	0.135
Relative estimation failure [mean ± std. dev., %]	6.97 (±5.3)	7.96 (±5.5)	0.099

## Data Availability

Data will be available after the manuscript was accepted.

## References

[1] Trudinger B.J., Stevens D., Connelly A., Hales J.R.S., Alexander G., Bradley L., Fawcett A., Thompson R.S. (1987). Umbilical artery flow velocity waveforms and placental resistance: The effects of embolization of the umbilical circulation. Am. J. Obstet. Gynecol..

[2] Alfirevic Z., Stampalija T., Dowswell T. (2017). Fetal and umbilical Doppler ultrasound in high-risk pregnancies. Cochrane Database Syst. Rev..

[3] Hecher K., Campbell S., Doyle P., Harrington K., Nicolaides K. (1995). Assessment of Fetal Compromise by Doppler Ultrasound Investigation of the Fetal Circulation. Circulation.

[4] Kehl S., Dötsch J., Hecher K., Schlembach D., Schmitz D., Stepan H., Gembruch U. (2017). Intrauterine Growth Restriction. Guideline of the German Society of Gynecology and Obstetrics (S2k-Level, AWMF Registry No. 015/080, October 2016). Geburtshilfe Frauenheilkd..

[5] Vayssière C., Sentilhes L., Ego A., Bernard C., Cambourieu D., Flamant C., Gascoin G., Gaudineau A., Grangé G., Houfflin-Debarge V. (2015). Fetal growth restriction and intra-uterine growth restriction: Guidelines for clinical practice from the French College of Gynaecologists and Obstetricians. Eur. J. Obstet. Gynecol. Reprod. Biol..

[6] Lausman A., Kingdom J., Gagnon R., Basso M., Bos H., Crane J., Davies G., Delisle M.-F., Hudon L., Menticoglou S. (2013). Intrauterine Growth Restriction: Screening, Diagnosis, and Management. J. Obstet. Gynaecol. Can..

[7] (2017). Management of Breech Presentation. BJOG Int. J. Obstet. Gynaecol..

[8] McCowan L.M., Figueras F., Anderson N.H. (2018). Evidence-based national guidelines for the management of suspected fetal growth restriction: Comparison, consensus, and controversy. Am. J. Obstet. Gynecol..

[9] Morris R., Say R., Robson S., Kleijnen J., Khan K.S. (2012). Systematic review and meta-analysis of middle cerebral artery Doppler to predict perinatal wellbeing. Eur. J. Obstet. Gynecol. Reprod. Biol..

[10] Heidweiller-Schreurs C.A.V., De Boer M.A., Heymans M., Schoonmade L.J., Bossuyt P.M.M., Mol B.W.J., De Groot C.J.M., Bax C.J. (2018). Prognostic accuracy of cerebroplacental ratio and middle cerebral artery Doppler for adverse perinatal outcome: Systematic review and meta-analysis. Ultrasound Obstet. Gynecol..

[11] Fong B.F., Savelsbergh G.J., Van Geijn H.P., De Vries J.I. (2005). Does intra-uterine environment influence fetal head-position preference? A comparison between breech and cephalic presentation. Early Hum. Dev..

[12] Bader B., Graham D., Stinson S. (1987). Significance of ultrasound measurements of the head of the breech fetus. J. Ultrasound Med..

[13] Melamed N., Yogev Y., Danon D., Mashiach R., Meizner I., Ben-Haroush A. (2010). Sonographic estimation of fetal head circumference: How accurate are we?. Ultrasound Obstet. Gynecol..

[14] Lau T.K., Leung T.Y., Lo K.W.K., Fok W.Y., Rogers M.S. (2000). Effect of external cephalic version at term on fetal circulation. Am. J. Obstet. Gynecol..

[15] McNamara J.M., Odibo A.O., Macones G.A., Cahill A.G. (2011). The Effect of Breech Presentation on the Accuracy of Estimated Fetal Weight. Am. J. Perinatol..

[16] Dammer U., Goecke T.W., Voigt F., Schmid M., Mayr A., Schild R.L., Beckmann M.W., Faschingbauer F. (2012). Sonographic weight estimation in fetuses with breech presentation. Arch. Gynecol. Obstet..

[17] Melamed N., Ben-Haroush A., Meizner I., Mashiach R., Yogev Y., Pardo J. (2011). Accuracy of sonographic fetal weight estimation: A matter of presentation. Ultrasound Obstet. Gynecol..

[18] Shmueli A., Aviram A., Bardin R., Wiznitzer A., Chen R., Gabbay-Benziv R. (2017). Effect of fetal presentation on sonographic estimation of fetal weight according to different formulas. Int. J. Gynecol. Obstet..

[19] Leung T.Y., Fok W.Y., Sahota D.S., Chan L.W., Lau T.K. (2004). External cephalic version induced fetal cerebral and umbilical blood flow changes are related to the amount of pressure exerted. BJOG Int. J. Obstet. Gynaecol..

[20] ACOG Mode of Term Singleton Breech Delivery—ACOG [Internet]. 2018 [Cited 26 October 2019]. https://www.acog.org/Clinical-Guidance-and-Publications/Committee-Opsinions/Committee-on-Obstetric-Practice/Mode-of-Term-Singleton-Breech-Delivery?IsMobileSet=false.

[21] Milner J., Arezina J. (2018). The accuracy of ultrasound estimation of fetal weight in comparison to birth weight: A systematic review. Ultrasound.

[22] Kotaska A., Menticoglou S., Gagnon R., Farine D., Basso M., Bos H., Delisle M.-F., Grabowska K., Hudon L., Mundle W. (2009). Vaginal delivery of breech presentation. Int. J. Gynecol. Obstet..

[23] Louwen F., Daviss B.-A., Johnson K.C., Reitter A. (2017). Does breech delivery in an upright position instead of on the back improve outcomes and avoid cesareans?. Int. J. Gynecol. Obstet..

[24] Jennewein L., Kielland-Kaisen U., Paul B., Möllmann C.J., Klemt A.-S., Schulze S., Bock N., Schaarschmidt W., Brüggmann D., Louwen F. (2018). Maternal and neonatal outcome after vaginal breech delivery at term of children weighing more or less than 3.8 kg: A FRABAT prospective cohort study. PLoS ONE.

